# A cross-sectional study on oral health-care habits and oral-health-related quality-of-life in marginalized persons in Copenhagen

**DOI:** 10.1080/00016357.2023.2282648

**Published:** 2024-03-22

**Authors:** Esben Boeskov Øzhayat, Ashraf Elmongy, Lene Tanderup, Sine Lykke Bordorff, Henrik Thiesen

**Affiliations:** a Section of community dentistry, department of Odontology, University of copenhagen, copenhagen, denmark; b Healthteam for the Homeless, copenhagen, center for Marginalized Adults and Families, copenhagen city Social Services, copenhagen, denmark

**Keywords:** Vulnerable populations, homeless persons, oral health, Denmark, quality of life

## Abstract

**Objective:**

The aim of this cross-sectional study was to investigate the oral-health-related quality-of-life (OHRQoL) and oral health-care habits in a population of marginalized persons in Copenhagen

**Materials and Methods:**

Patients attending a dental clinic for marginalized persons filled in the 14-item version of the oral-health-impact profile (OHIP-14) regarding their OHRQoL and a questionnaire on their oral health-care habits. Age, gender, smoking habits, need for general and oral health-care, and living conditions were further registered.

**Results:**

Of the 212 participants, 72% had not visited a dental clinic within the past two years and in 68% of the cases, the last dental visit was related to dental treatment. Tooth brushing at least once a day was reported by 93%. The mean OHIP-14 score in the participants was 24.9 (SD: 13.6). The most frequent problems were pain, chewing difficulties, being self-conscious, tense, and embarrassed as well as affected life. The mean OHIP-14 score was significantly higher in participants in need of general health-care (29.5, SD: 12.2) than in participants not in need of general health-care (22.8, SD: 13.9). The same applied to participants referred for dental treatment (26.1, SD: 12.7) compared to participants not being referred (20.2, SD: 15.9).

**Conclusions:**

The OHRQoL is poor in the population with pain, chewing difficulties and aesthetic issues as the most prominent problems. The participants had low and treatment-oriented use of the dental care system. This indicates a high need for dental care in the population with a focus on including them in the dental care system.

## Introduction

Oral health is an integral part of general health and is highly related to social functioning and quality-of-life [1, 2]. Poor oral health thus can have a negative impact on a person’s life. In Denmark, poor oral health has become less frequent during the last decades [3], but the distribution of the phenomenon is not equal in the population. The social inequality in oral health is thus persisting, and is higher than in many other European countries [4]. The general population has few problems with their teeth and mouth, whereas there are reports of high frequency of poor oral health in socially marginalized persons [5, 6]. Marginalized persons have many both social and health issues including unemployment, mental health issues, and shorter life expectancy. They live on the edge of society battling with among other things addictions and homelessness. Unfortunately, this population uses the oral health-care system to a lower degree than the general population [3, 5]. This is what is referred to as the ‘inverse care law’ and makes the problem even greater. In addition, it has been described how this group, due to their poor oral health, might be stigmatized and excluded from the health-and social systems in society including the labor market [7].

For this reason, efforts have been implemented to improve oral health in the marginalized group. So far, the experiences are not great, with the main challenges being poor oral hygiene and poor use of the dental care system. In Denmark, it has thus been a priority to incorporate these citizens into the dental care system in order to treat them and initiate preventive measures. Unfortunately, the initiatives to incorporate them into the system thus far have failed [8]. An important challenge is the competing health and social issues found in this group [9], which often results in low prioritization of oral health.

If we are to have success in including marginalized persons in the dental care system, it is crucial to know what types of oral problems they face, how it affects them, and what their oral health-care habits are. In this regard, oral-health-related quality-of-life (OHRQoL) seems as a highly relevant parameter to investigate, as it can indicate what challenges the population encounters, and how much the problems impact their lives. Some studies have investigated OHRQoL and oral health-care habits in marginalized populations [6, 10, 11], but there is still much we do not know about this vulnerable group. One of the reasons for the lack of knowledge is the difficulties in including the group in epidemiological studies [12, 13].

We therefore conducted a study with the aim to describe the OHRQoL and oral health-care habits in the most marginalized persons in Copenhagen. The knowledge from this study will be highly relevant when preparing for inclusion of this group in the dental care system or creating other initiatives to improve oral health in marginalized populations, such as prevention programs and actual dental care.

## Material and methods

### Participants and setting

Until 2020, three dental clinics offered free dental services for the most marginalized persons in Copenhagen. The most marginalized persons are people living in the street or in temporary housing who only with great difficulty can use the health-care system including dental care. One of these clinics was part of a more general health-care offer at the Sundholm health-care center in the Southern part of Copenhagen. The clinic had easy access to the target group and a wish to measure OHRQoL in their patients, which made it a good study site. The clinic was in use from 2018–2020 and was staffed with a single dental hygienist, who made clinical examinations including diagnostics of oral diseases and performed preventive dental care. If the dental hygienist diagnosed oral diseases with a need for treatment, the patient was referred to one of the two other clinics offering free dental care for marginalized persons, as they were staffed with dentists. In the period, 212 patients had contact to the clinic and as part of the initial recordings, all patients were asked to fill in a questionnaire on their oral health-care habits and OHRQoL and were potential participants in the study. There were no other inclusion and exclusion criteria applied to be included and the sample was thus based on convenience. The patients willingly participated but did not sign an informed consent. They were pseudo-anonymized, and the data were handled without the researchers knowing the identity of the patient. The project is approved by the Danish Data Protection Agency (514-0658/21-3000) and the local ethical committee at the Faculty of Health, University of Copenhagen (504-0302/22-5000).

### Methods

In this cross-sectional study, oral health-care habits were investigated by asking the participants about their use of the dental care system and their daily oral hygiene. Use of the dental care system was evaluated by having the participant answer when they last had contact with the system (less than 6 months, 6–12 months, more than 1 year – but less than 2, 2–5 years, more than 5 years). For analytical purposes, the following categories were used: contact within 2 years, contact within 2–5 years, and no contact for more than 5 years. The reason for their last visit was also registered (consultation, pain or other oral-related problem, planned treatment, regular check-up, other). The following categories were used for analytical purposes: consultation/regular check-ups, treatment, other reason. The oral hygiene was evaluated by having the participants answer how often they brushed their teeth (never, once in a while – not every week, several times a week – not every day, once a day, two or more times a day). For analytical purposes, the following categories were used: never, once in a while/not every day, daily brushing. If they used toothpaste when brushing was also registered (yes, once in a while, no). The questions on habits were developed by the dental hygienist in the clinic and were not validated before use.

The OHRQoL was measured with the 14-item oral-health-impact profile (OHIP-14), which consists of 14 questions related to problems in the oral region [14]. The participants answered how often each problem had occurred during the past month on a scale with six choices and correspondent scores: *very often* (4), *fairly often* (3), *occasionally* (2), *hardly ever* (1), *never* (0) or *don’t know*. To calculate an overall OHIP-14 score for each participant, the scores from the 14 answers are added, thereby giving a score between 0 and 56. A higher score thus indicates a worse quality-of-life. The OHIP-14 score was further dichotomized using the mean OHIP-14 score. OHIP-14 items with score 3 and 4 were considered as experienced problems [15]. The frequency of problems was calculated and reported in percentage of the population having the problems.

Besides the oral health-care habits and OHRQoL, the participant’s gender and age were registered. Due to the relatively low number of participants, age was divided into two categories based on the mean age. It was also registered if they smoked (yes/no), if they currently needed general health-care services in the clinic (yes/no), and if they were referred to a dental clinic for treatment (yes/no). Their living condition was registered according to the European Typology of Homelessness (ETHOS) classification [16] and the following categories were used: homeless, temporary housing, housing offer for addicts or mentally ill, own housing.

### Analyses

Analyses were performed using the Statistical Package for the Social Science (SPSS) version 28 and the significance level was 0.05. Descriptive analyses were used to describe the oral health-care habits and quality-of-life in the population. The frequency of problems was illustrated with bar plots. The OHIP-14 score was normally distributed according to the test of skewness and kurtosis and the Kolmogorov-Smirnov test. Bivariate analyses between the OHIP-14 score and the categorical variables for age, gender, living condition, referral, contact to the dentist, and tooth brushing habits were performed using t-tests and ANOVA with post hoc analysis. The difference in frequency of problems between participants referred to dental treatment and participants not referred to treatment was specifically tested, as this gives valuable knowledge on what dentists can expect when receiving such patients in their clinic. Logistic regression analysis was performed with the categorical OHIP-14 variable as an outcome and the variables used in the bivariate analyses as explanatory.

## Results

All of the 212 citizens had age and gender registered and almost all had living conditions, smoking, the need of health-care, and referral status registered (Table 1). Oral health-care habits and OHRQoL were reported in 186 (88%) and 182 (86%) respectively. Of the participants reporting OHRQoL, none had missing values in any of the OHIP-14 items. The mean age of the participants was 44.40 (SD 12.36) and the distribution of the categorical variables is shown in Table 1. Most participants were male, living in temporary housings, were smokers, and did not need general health-care. The participants most often were referred to dental treatment, had not seen a dentist in more than two years, had treatment as their last contact with a dentist, and brushed their teeth frequently with toothpaste. Drop-out analyses between participants filling in and not filling in the OHIP-14 showed no statistical differences in the distribution in any of the explanatory variables.

**Table 1:** Distribution of categorical variables.

Variable	Distribution
Age (*n* = 212)	
20–44	49.5 %
45–69	50.5 %
Gender (*n* = 212)	
Female	62 (29.2 %)
Male	150 (70.8 %)
Living condition (*n* = 201)	
Homeless	22 (10.9 %)
Temporary housing	125 (62.2 %)
Housing offer for addicts or mentally	16 (8.0 %)
ill	
Own housing	38 (18.9 %)
Smoking (*n* = 201)	
Yes	169 (84.1 %)
No	32 (15.9 %)
Need of health-care (*n* = 203)	
Yes	60 (29.6 %)
No	143 (70.4 %)
Referred to oral treatment (*n* = 205)	
Yes	159 (77.6 %)
No	46 (22.4 %)
Last dental contact (*n* = 186)	
Within 2 years	52 (28.0 %)
2–5 years	88 (47.3 %)
More than 5 years	46 (24.7 %)
Reason for last dental contact (*n* = 186)	
Consultation/regular check-up	38 (20.4 %)
Treatment	127 (68.3 %)
Other	21 (11.3 %)
Frequency of tooth brushing (*n* = 186)	
Never	12 (6.5 %)
Once in a while/not every day	59 (31.7 %)
Daily brushing	115 (61.8 %)
Use of toothpaste (*n* = 186)	
Yes	167 (89.8 %)
Once in a while	8 (4.3 %)
No	11 (5.9 %)
OHIP-14 score (*n* = 182)	
0–24	90 (49.5 %)
25–56	92 (50.5 %)

The mean OHIP-14 score in the total population was 24.9 (SD: 13.6). The bivariate analyses showed that most of the groups did not differ significantly in the mean OHIP-14 score (Table 2). Needing general health-care and being referred to dental treatment was, however, associated with significantly higher OHIP-14 scores compared to not needing general health-care and not being referred to dental treatment. The logistic regression analysis showed that participants needing general health-care had an odds ratio of 2.4 (CI 1.1–4.7) for being in the high OHIP-14 score group compared to participants not needing general health-care (Table 3). None of the other odds ratios were significant.

**Table 2:** Associations between explanatory variables and mean OHIP-14 score.

Variable	Mean OHIP-14 score (SD)
Age (*n* = 212)	
20–44	24.8 (13.0)
45–69	25.0 (14.3)
Gender (*n* = 212)	
Female	26.9 (15.0)
Male	24.1 (13.0)
Living condition (*n* = 201)	
Homeless	24.5 (12.5)
Temporary housing	25.8 (13.6)
Housing offer for addicts or mentally	23.2 (13.8)
ill	
Own housing	24.4 (14.1)
Need of health-care (*n* = 203)	
No	22.8 (13.9)
Yes	29.5 (12.2)*
Referred to oral treatment (*n* = 205)	
No	20.2 (15.9)
Yes	26.1 (12.7)*
Last dental contact (*n* = 186)	
within 2 years	27.1 (15.1)
2–5 years	24.0 (13.6)
more than 5 years	24.4 (12.0)
Frequency of tooth brushing (*n* = 186)	
daily brushing	23.7 (13.9)
once in a while/not every day	27.3 (13.2)
never	24.7 13.4)

* *p* < 0.05.

**Table 3:** Logistic regression analyses.

Variable	Frequency (%) high OHIP-14 score	Unadjusted OR (95 % CI)	*P*	Adjusted OR (95 % CI)	*P*
Age (*n* = 212)					
20–44	47 (50.0 %)	Ref.		Ref.	
45–69	45 (51.1 %)	1.05 (0.6–1.9)	0.88	1.2 (0.6–2.2)	0.63
Gender (*n* = 212)					
Female	30 (57.7 %)	Ref.		Ref.	
Male	62 (47.7 %)	0.7 (0.3–1.3)	0.22	0.8 (0.4–1.5)	0.41
Living condition					
(*n* = 201)					
Homeless	8 (53.3 %)	Ref.		Ref.	
Temporary housing	63 (54.3 %)	(0.4–3.1)	0.94	1.2 (0.4–3.9)	0.75
Housing offer for	5 (35.7 %)	0.5 (0.1–2.2)	0.34	0.6 (0.1–3.0)	0.57
addicts or					
mentally ill					
Own housing	16 (48.5 %)	0.8 (0.2–2.8)	0.76	0.8 (0.2–3.2)	0.77
Need of health-care					
(*n* = 203)					
No	56 (43.8 %)	Ref.		Ref.	
Yes	30 (62.5 %)	2.1 (1.1–4.2)	0.03	2.2 (1.1–4.7)	0.04
Referred to oral					
treatment					
(*n* = 205)					
No	17 (40.5 %)	Ref.		Ref.	
Yes	71 (52.2 %)	1.6 (0.8–3.2)	0.19	1.2 (0.6–2.6)	0.62
Last dental contact					
(*n* = 186)					
within 2 years	27 (52.9 %)	Ref.		Ref.	
2–5 years	42 (48.3 %)	0.8 (04–1.7)	0.60	0.8 (0.4–1.6)	0.49
more than 5 years	23 (53.5 %)	1.0 (0.5–2.3)	0.96	1.0 (0.4–2.5)	0.95
Frequency of tooth					
brushing (*n* = 186)					
daily brushing	55 (49.1 %)	Ref.		Ref.	
once in a while/	32 (55.2 %)	1.3 (0.7–2.4)	0.45	1.2 (0.6–2.4)	0.68
not every day					
never	5 (41.7 %)	0.7 (0.2–2.5)	0.63	0.7 (0.2–2.7)	0.61

Adjusted for all explanatory variables. OR = odds ratio, CI = confidence interval.

**Figure 1:**
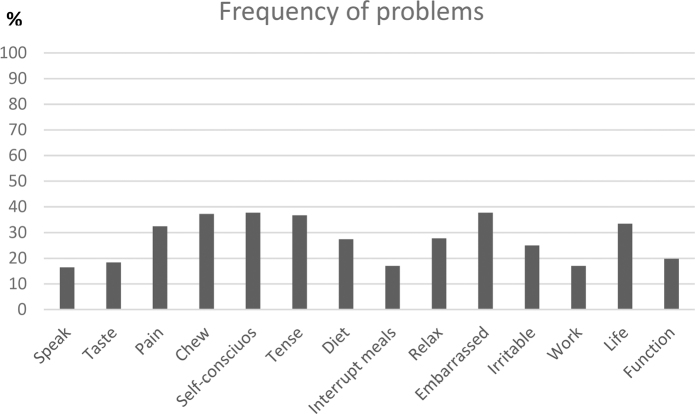
Frequency (%) of the OHIP-14 items being reported as a problem in the study population.

**Figure 2:**
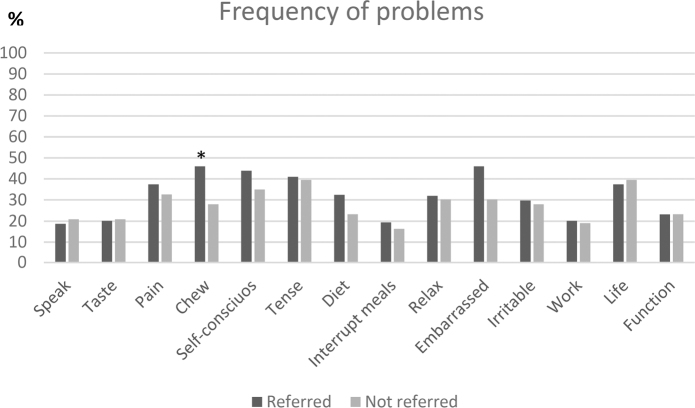
Frequency (%) of the OHIP-14 items being reported as a problem in participants being reffered and not being reffered for dental treatment.

In the study population, it was found that 73.3% had at least one problem. Figure 1 shows the frequency of experienced problems in the study population for each item. It is seen that pain, chewing difficulties, being self-conscious, tense and embarrassed is the most frequent problems alongside impact on life in general. When comparing participants who were referred and not referred to dental treatment, it is seen that participants who were referred to treatment in most items more frequently had problems (Figure 2). This was especially pronounced for chewing difficulties (*p* = 0.04), being self-conscious (*p* = 0.29), and being embarrassed (*p* = 0.07).

## Discussion

This study investigated the OHRQoL and oral health-care habits in a marginalized population in Copenhagen. The main finding is poor OHRQoL in the group with the most frequent problems being pain, chewing difficulties and aesthetic issues. The participants had low, treatment-oriented use of the dental care system and a high frequency of tooth brushing. Furthermore, participants were often referred for dental treatment. Referral for dental treatment and needing general health-care were associated with poorer OHRQoL.

The poor OHRQoL in the participants is not surprising and similar levels of OHIP-14 scores have been reported in other studies [5, 6, 17]. This does, however, not make the finding irrelevant. The more evidence for the oral-related problems in vulnerable people we have and the more knowledge of the reasons for this, the more realistic it is to change the situation and achieve more equality in oral health. Other studies investigating the experienced problems using OHIP-14 in marginalized people have reported the same problems as being the most frequent as the ones in this study [5, 6, 11]. This indicates that oral pain, having trouble eating your food, and aesthetic issues regarding your teeth affects the life in this group to a large degree. From our study, it is noteworthy that the participants that were referred for dental treatment, in general, had more problems related specifically to chewing difficulties and aesthetic problems. This is important knowledge for the clinicians they are referred to. Luckily, it has been found that oral treatment with relatively simple measures can increase marginalized people’s OHRQoL significantly [5].

The association between OHRQoL and the explanatory variables used was very limited. This implies either no association between the variables or to small power to find significant results, which both could be the case in our study. The explanatory variables in our study are relevant, but other relevant variables could have been included. It is especially a limitation that the clinical oral status of the participants was not included since it has been found that high DMFT and periodontal problems can be associated with poor OHRQoL in homeless people [17]. Also, other socioeconomic variables such as ethnicity and length of stay in Denmark could have been relevant to include. Further, even though the OHIP-14 questionnaire is a validated measure, the questions on oral-health habits were not and could have been more in line with international standards, to better compare to other studies. The power in our study is also limited due to the small population size, at least in some of the subgroups. The non-significant findings are, however, not useless. It is interesting that no matter your living condition, gender, age and oral health-care habits, being marginalized is associated with poor OHRQoL. This implies that the target group in general has great oral problems, and it can be difficult to predict which citizens have oral problems based on their oral heal-care habits. This is important knowledge for people working with marginalized persons. The association between needing general health-care and poor OHRQoL indicates that a poor general health status also is reflected in oral health. It has been described how this group has a broad spectrum of health and social issues [9], which naturally affect their lives. Seen as the view on life also influences the OHRQoL [18], it is thus not surprising that participants with general health problems report worse OHRQoL.

The oral health-care habits investigated in our study is relevant, even though they could have been supplemented with data on their diet. It was not surprising that few participants had contact with the dental care system within the last two years and that the contact most often was related to treatment and not prevention. Other studies have also shown a limited use of the dental care system and several barriers to this in the most marginalized people [19]. Even though other studies also have reported frequent tooth brushing in marginalized groups [5, 20], it was rather surprising that the group reported frequent tooth brushing and use of toothpaste. The reason for the high frequency of tooth brushing might be true, but it might also reflect the societal expectation that you should report that you brush your teeth; otherwise, there is a risk of being stigmatized.

An important aspect to discuss is the representativeness of our results. Because the participants found their way to the health clinic, they might be considered some of the more resourceful marginalized people, which would imply better oral health than the entire target population. On the other hand, an overestimation of problems might be the case as the recruitment was done in a dental care clinic and the participants could have visited the clinic due to oral problems. Further, it is important to note that the setting is rather local, which could question the generalizability of our results. It seems, however, from other studies in other settings, that many of the same issues and challenges are found in marginalized people.

We hope that the findings from our study will help to put focus on the troubles of marginalized persons including their challenges in regard to oral health. Quality-of-life was highly affected and improvement in oral health is thus very much needed in the population. Given our results are valid, it seems that even though improvement in daily oral health-care is desirable, increasing the use of the dental care system and receiving oral rehabilitation and preventive measures is an obvious place to start. In this regard, it is relevant that a newly published study has found that an oral health motivation intervention in marginalized people not only led to an increase in the use of the dental care system but also had an effect on the proximity to the labor market [21]. To improve oral health-care habits in marginalized people, it has been suggested to improve the awareness and knowledge in the population and implement supportive systems focusing on holistic and flexible care, outreach, interdisciplinary teams, and effective communication [22].
